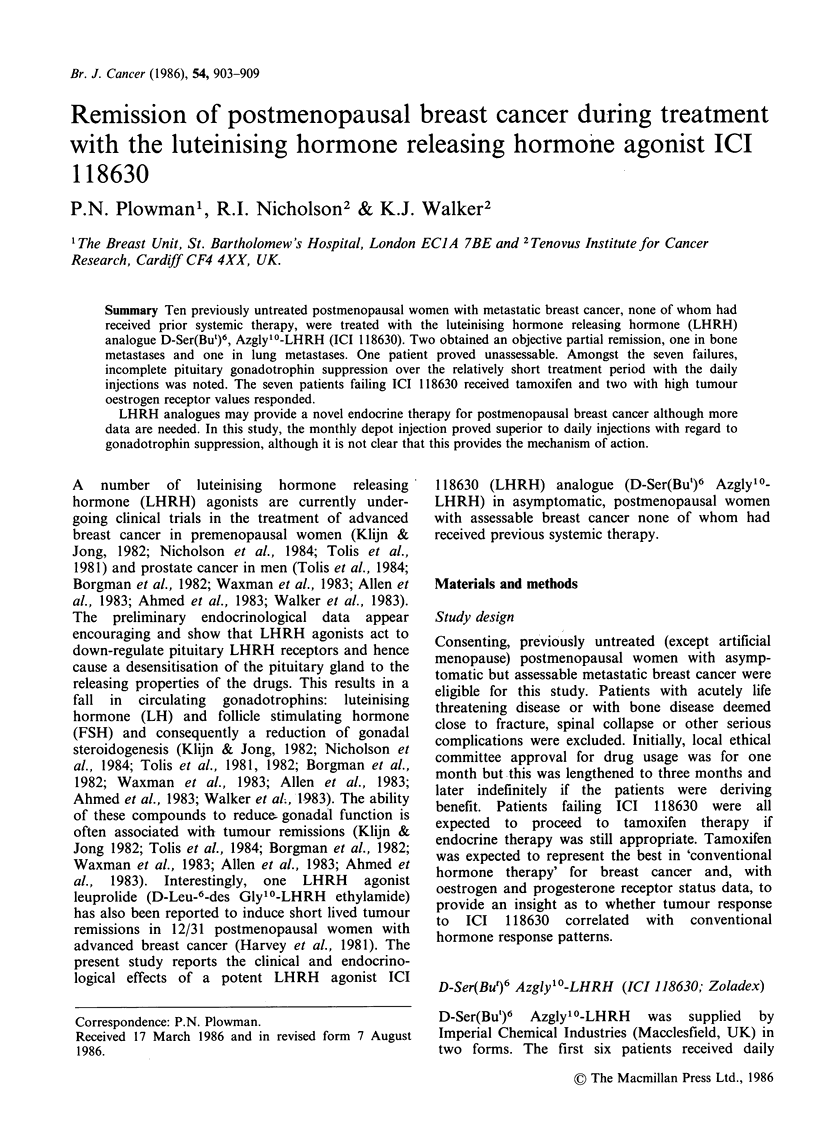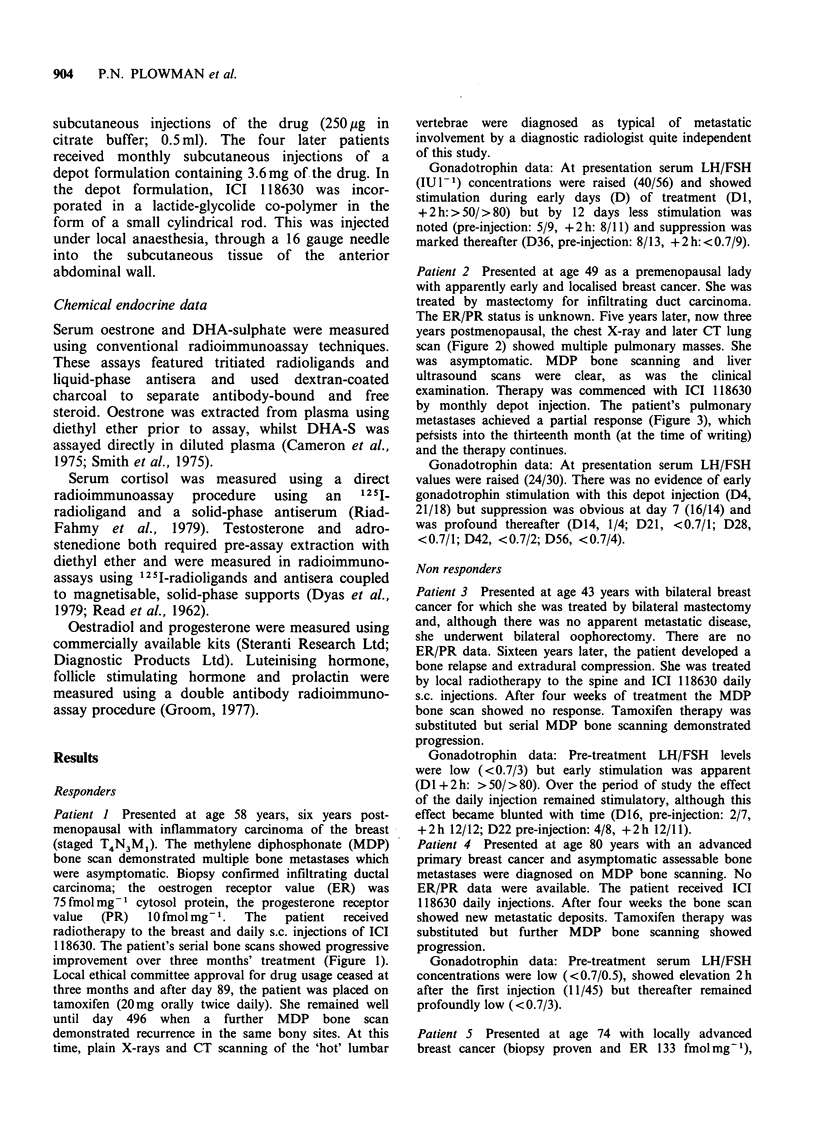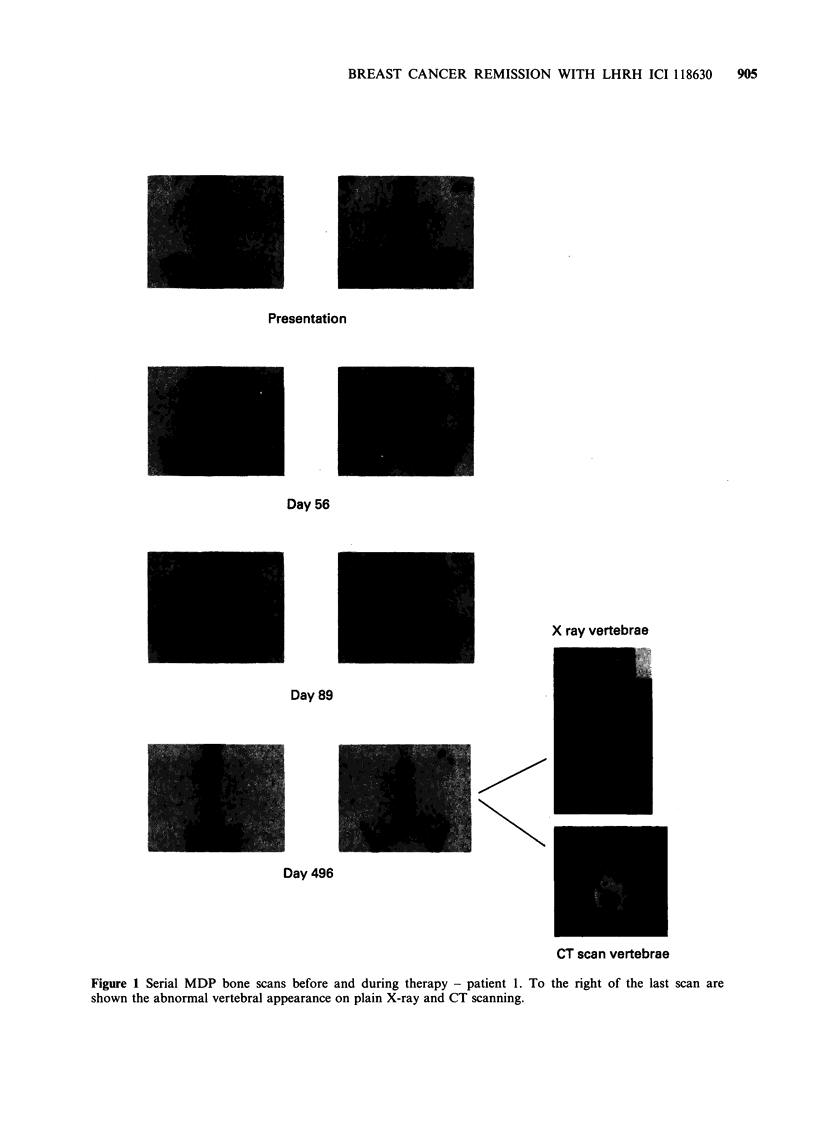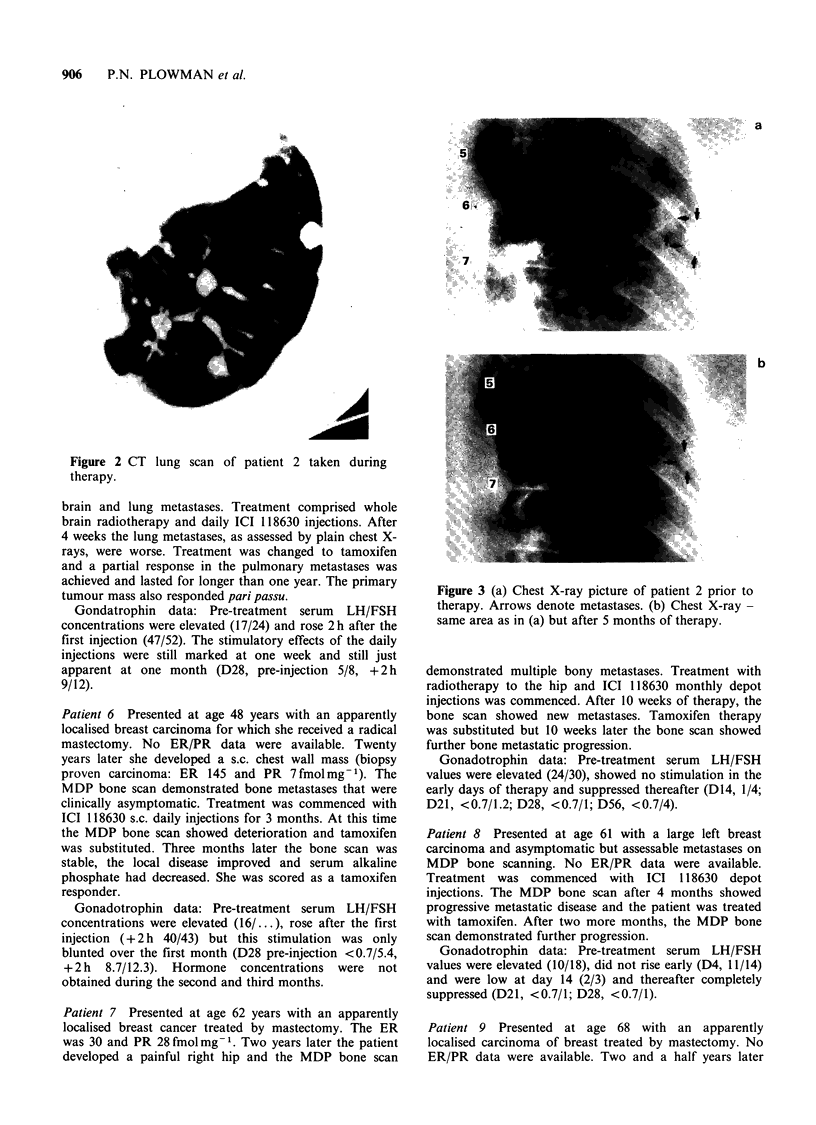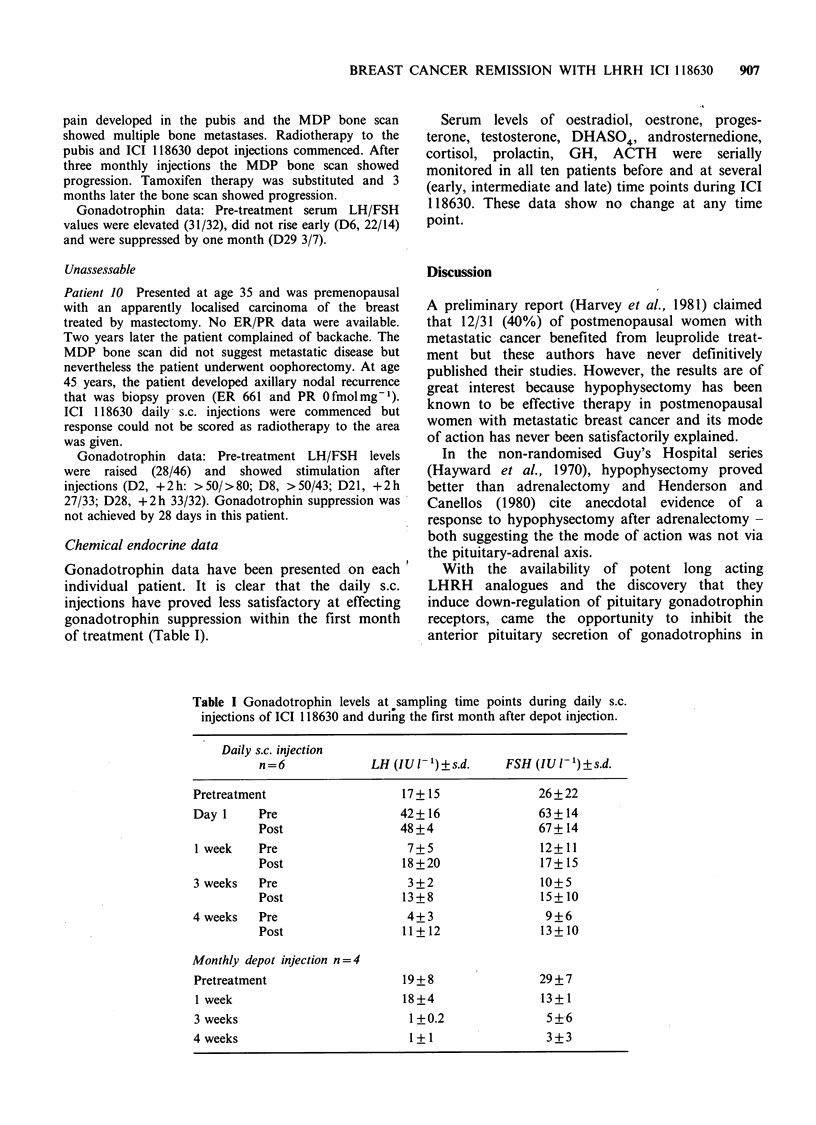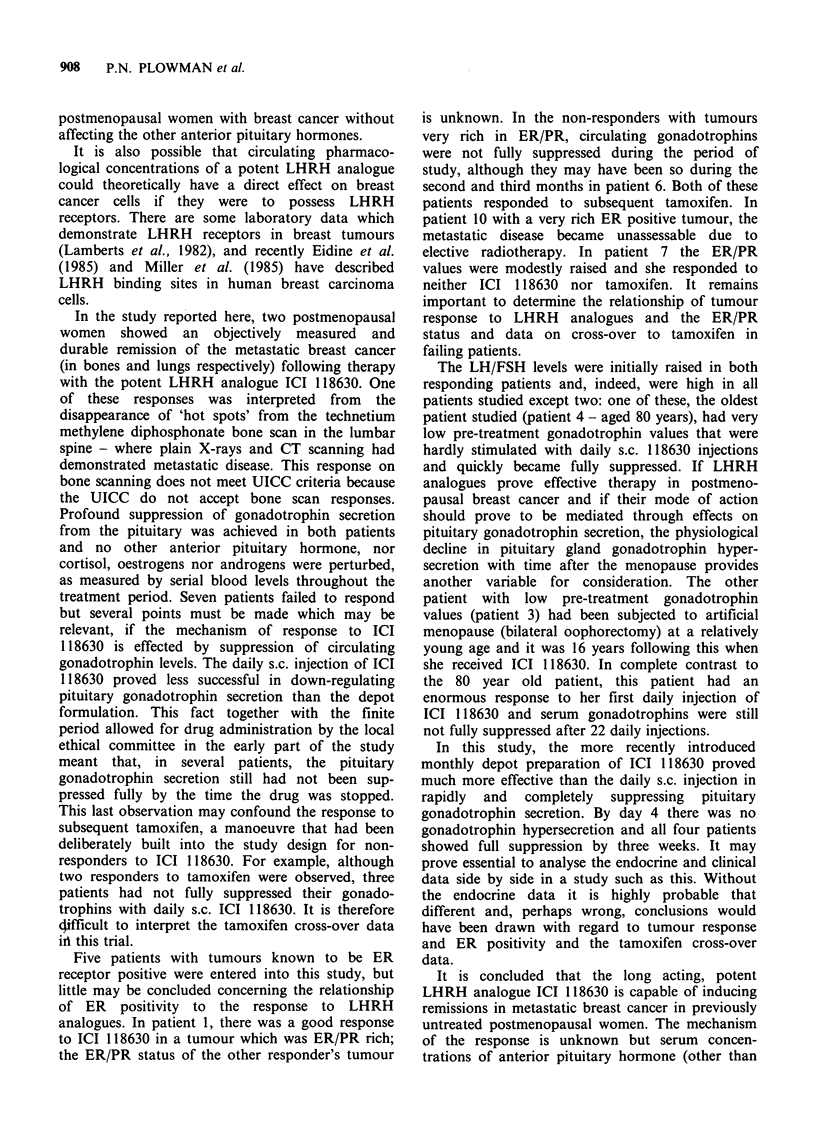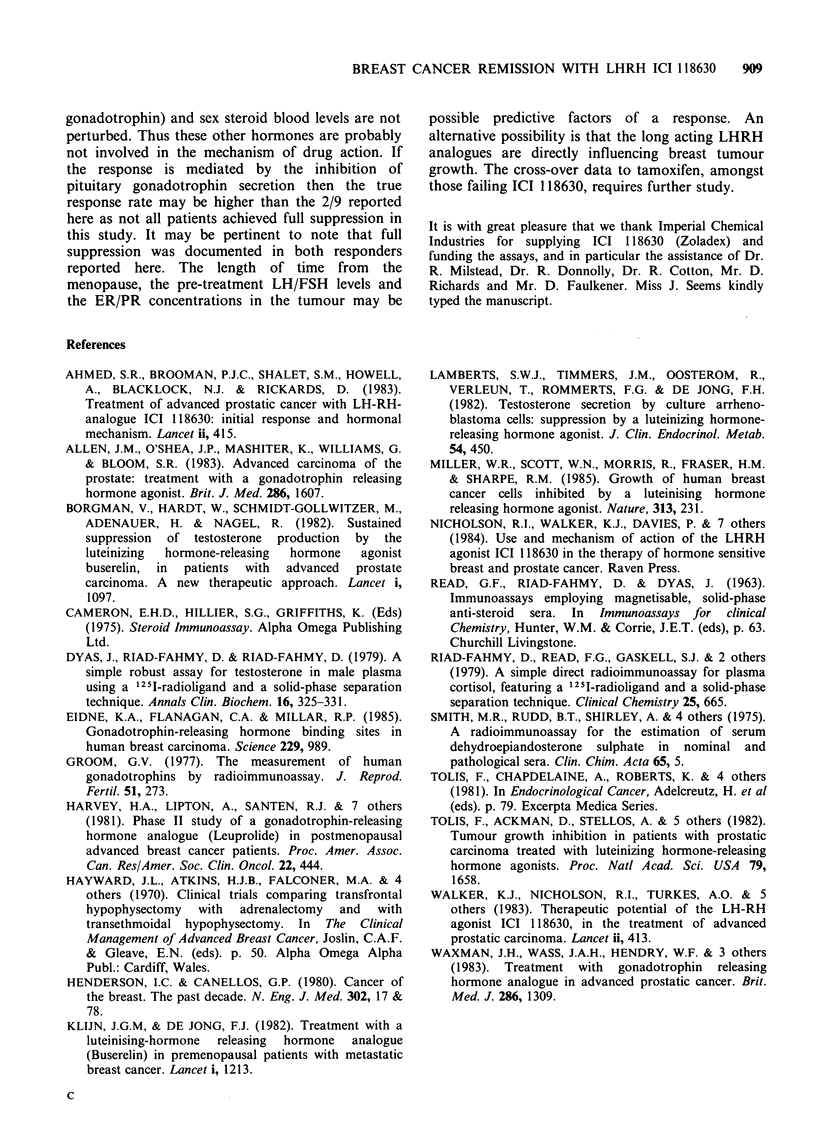# Remission of postmenopausal breast cancer during treatment with the luteinising hormone releasing hormone agonist ICI 118630.

**DOI:** 10.1038/bjc.1986.260

**Published:** 1986-12

**Authors:** P. N. Plowman, R. I. Nicholson, K. J. Walker

## Abstract

**Images:**


*Br. J. Cancer* (1986), **54**, 903–909

### Remission of postmenopausal breast cancer during treatment with the luteinising hormone releasing hormone agonist ICI 118630

P.N. Plowman[1], R.I. Nicholson[2] & K.J. Walker[2]

[1]*The Breast Unit, St. Bartholomew's Hospital, London EC1A 7BE and* [2]*Tenovus Institute for Cancer Research, Cardiff CF4 4XX, UK.*

**Summary** Ten previously untreated postmenopausal women with metastatic breast cancer, none of whom had received prior systemic therapy, were treated with the luteinising hormone releasing hormone (LHRH) analogue D-Ser(Bu$^t$)$^6$, Azgly$^{10}$-LHRH (ICI 118630). Two obtained an objective partial remission, one in bone metastases and one in lung metastases. One patient proved unassessable. Amongst the seven failures, incomplete pituitary gonadotrophin suppression over the relatively short treatment period with the daily injections was noted. The seven patients failing ICI 118630 received tamoxifen and two with high tumour oestrogen receptor values responded.

LHRH analogues may provide a novel endocrine therapy for postmenopausal breast cancer although more data are needed. In this study, the monthly depot injection proved superior to daily injections with regard to gonadotrophin suppression, although it is not clear that this provides the mechanism of action.

A number of luteinising hormone releasing hormone (LHRH) agonists are currently undergoing clinical trials in the treatment of advanced breast cancer in premenopausal women (Klijn & Jong, 1982; Nicholson *et al.*, 1984; Tolis *et al.*, 1981) and prostate cancer in men (Tolis *et al.*, 1984; Borgman *et al.*, 1982; Waxman *et al.*, 1983; Allen *et al.*, 1983; Ahmed *et al.*, 1983; Walker *et al.*, 1983). The preliminary endocrinological data appear encouraging and show that LHRH agonists act to down-regulate pituitary LHRH receptors and hence cause a desensitisation of the pituitary gland to the releasing properties of the drugs. This results in a fall in circulating gonadotrophins: luteinising hormone (LH) and follicle stimulating hormone (FSH) and consequently a reduction of gonadal steroidogenesis (Klijn & Jong, 1982; Nicholson *et al.*, 1984; Tolis *et al.*, 1981, 1982; Borgman *et al.*, 1982; Waxman *et al.*, 1983; Allen *et al.*, 1983; Ahmed *et al.*, 1983; Walker *et al.*, 1983). The ability of these compounds to reduce gonadal function is often associated with tumour remissions (Klijn & Jong 1982; Tolis *et al.*, 1984; Borgman *et al.*, 1982; Waxman *et al.*, 1983; Allen *et al.*, 1983; Ahmed *et al.*, 1983). Interestingly, one LHRH agonist leuprolide (D-Leu-$^6$-des Gly$^{10}$-LHRH ethylamide) has also been reported to induce short lived tumour remissions in 12/31 postmenopausal women with advanced breast cancer (Harvey *et al.*, 1981). The present study reports the clinical and endocrinological effects of a potent LHRH agonist ICI 118630 (LHRH) analogue (D-Ser(Bu$^t$)$^6$ Azgly$^{10}$-LHRH) in asymptomatic, postmenopausal women with assessable breast cancer none of whom had received previous systemic therapy.

Correspondence: P.N. Plowman.
Received 17 March 1986 and in revised form 7 August 1986.

### Materials and methods

### *Study design*

Consenting, previously untreated (except artificial menopause) postmenopausal women with asymptomatic but assessable metastatic breast cancer were eligible for this study. Patients with acutely life threatening disease or with bone disease deemed close to fracture, spinal collapse or other serious complications were excluded. Initially, local ethical committee approval for drug usage was for one month but this was lengthened to three months and later indefinitely if the patients were deriving benefit. Patients failing ICI 118630 were all expected to proceed to tamoxifen therapy if endocrine therapy was still appropriate. Tamoxifen was expected to represent the best in 'conventional hormone therapy' for breast cancer and, with oestrogen and progesterone receptor status data, to provide an insight as to whether tumour response to ICI 118630 correlated with conventional hormone response patterns.

### *D-Ser(Bu$^t$)$^6$ Azgly$^{10}$-LHRH (ICI 118630; Zoladex)*

D-Ser(Bu$^t$)$^6$ Azgly$^{10}$-LHRH was supplied by Imperial Chemical Industries (Macclesfield, UK) in two forms. The first six patients received daily

© The Macmillan Press Ltd., 1986

subcutaneous injections of the drug (250 $\mu$g in citrate buffer; 0.5 ml). The four later patients received monthly subcutaneous injections of a depot formulation containing 3.6 mg of the drug. In the depot formulation, ICI 118630 was incorporated in a lactide-glycolide co-polymer in the form of a small cylindrical rod. This was injected under local anaesthesia, through a 16 gauge needle into the subcutaneous tissue of the anterior abdominal wall.

*Chemical endocrine data*

Serum oestrone and DHA-sulphate were measured using conventional radioimmunoassay techniques. These assays featured tritiated radioligands and liquid-phase antisera and used dextran-coated charcoal to separate antibody-bound and free steroid. Oestrone was extracted from plasma using diethyl ether prior to assay, whilst DHA-S was assayed directly in diluted plasma (Cameron *et al.*, 1975; Smith *et al.*, 1975).

Serum cortisol was measured using a direct radioimmunoassay procedure using an $^{125}$I-radioligand and a solid-phase antiserum (Riad-Fahmy *et al.*, 1979). Testosterone and adrostenedione both required pre-assay extraction with diethyl ether and were measured in radioimmunoassays using $^{125}$I-radioligands and antisera coupled to magnetisable, solid-phase supports (Dyas *et al.*, 1979; Read *et al.*, 1962).

Oestradiol and progesterone were measured using commercially available kits (Steranti Research Ltd; Diagnostic Products Ltd). Luteinising hormone, follicle stimulating hormone and prolactin were measured using a double antibody radioimmunoassay procedure (Groom, 1977).

### Results

*Responders*

*Patient 1* Presented at age 58 years, six years postmenopausal with inflammatory carcinoma of the breast (staged $T_4N_3M_1$). The methylene diphosphonate (MDP) bone scan demonstrated multiple bone metastases which were asymptomatic. Biopsy confirmed infiltrating ductal carcinoma; the oestrogen receptor value (ER) was 75 fmol mg$^{-1}$ cytosol protein, the progesterone receptor value (PR) 10 fmol mg$^{-1}$. The patient received radiotherapy to the breast and daily s.c. injections of ICI 118630. The patient's serial bone scans showed progressive improvement over three months' treatment (Figure 1). Local ethical committee approval for drug usage ceased at three months and after day 89, the patient was placed on tamoxifen (20 mg orally twice daily). She remained well until day 496 when a further MDP bone scan demonstrated recurrence in the same bony sites. At this time, plain X-rays and CT scanning of the 'hot' lumbar vertebrae were diagnosed as typical of metastatic involvement by a diagnostic radiologist quite independent of this study.

Gonadotrophin data: At presentation serum LH/FSH (IU l$^{-1}$) concentrations were raised (40/56) and showed stimulation during early days (D) of treatment (D1, +2 h: >50/>80) but by 12 days less stimulation was noted (pre-injection: 5/9, +2 h: 8/11) and suppression was marked thereafter (D36, pre-injection: 8/13, +2 h: <0.7/9).

*Patient 2* Presented at age 49 as a premenopausal lady with apparently early and localised breast cancer. She was treated by mastectomy for infiltrating duct carcinoma. The ER/PR status is unknown. Five years later, now three years postmenopausal, the chest X-ray and later CT lung scan (Figure 2) showed multiple pulmonary masses. She was asymptomatic. MDP bone scanning and liver ultrasound scans were clear, as was the clinical examination. Therapy was commenced with ICI 118630 by monthly depot injection. The patient's pulmonary metastases achieved a partial response (Figure 3), which persists into the thirteenth month (at the time of writing) and the therapy continues.

Gonadotrophin data: At presentation serum LH/FSH values were raised (24/30). There was no evidence of early gonadotrophin stimulation with this depot injection (D4, 21/18) but suppression was obvious at day 7 (16/14) and was profound thereafter (D14, 1/4; D21, <0.7/1; D28, <0.7/1; D42, <0.7/2; D56, <0.7/4).

*Non responders*

*Patient 3* Presented at age 43 years with bilateral breast cancer for which she was treated by bilateral mastectomy and, although there was no apparent metastatic disease, she underwent bilateral oophorectomy. There are no ER/PR data. Sixteen years later, the patient developed a bone relapse and extradural compression. She was treated by local radiotherapy to the spine and ICI 118630 daily s.c. injections. After four weeks of treatment the MDP bone scan showed no response. Tamoxifen therapy was substituted but serial MDP bone scanning demonstrated progression.

Gonadotrophin data: Pre-treatment LH/FSH levels were low (<0.7/3) but early stimulation was apparent (D1+2 h: >50/>80). Over the period of study the effect of the daily injection remained stimulatory, although this effect became blunted with time (D16, pre-injection: 2/7, +2 h 12/12; D22 pre-injection: 4/8, +2 h 12/11).

*Patient 4* Presented at age 80 years with an advanced primary breast cancer and asymptomatic assessable bone metastases were diagnosed on MDP bone scanning. No ER/PR data were available. The patient received ICI 118630 daily injections. After four weeks the bone scan showed new metastatic deposits. Tamoxifen therapy was substituted but further MDP bone scanning showed progression.

Gonadotrophin data: Pre-treatment serum LH/FSH concentrations were low (<0.7/0.5), showed elevation 2 h after the first injection (11/45) but thereafter remained profoundly low (<0.7/3).

*Patient 5* Presented at age 74 with locally advanced breast cancer (biopsy proven and ER 133 fmol mg$^{-1}$),

**Figure 1** Serial MDP bone scans before and during therapy – patient 1. To the right of the last scan are shown the abnormal vertebral appearance on plain X-ray and CT scanning.

**Figure 2** CT lung scan of patient 2 taken during therapy.

brain and lung metastases. Treatment comprised whole brain radiotherapy and daily ICI 118630 injections. After 4 weeks the lung metastases, as assessed by plain chest X-rays, were worse. Treatment was changed to tamoxifen and a partial response in the pulmonary metastases was achieved and lasted for longer than one year. The primary tumour mass also responded *pari passu*.

Gondatrophin data: Pre-treatment serum LH/FSH concentrations were elevated (17/24) and rose 2 h after the first injection (47/52). The stimulatory effects of the daily injections were still marked at one week and still just apparent at one month (D28, pre-injection 5/8, +2 h 9/12).

*Patient 6* Presented at age 48 years with an apparently localised breast carcinoma for which she received a radical mastectomy. No ER/PR data were available. Twenty years later she developed a s.c. chest wall mass (biopsy proven carcinoma: ER 145 and PR 7 fmol mg$^{-1}$). The MDP bone scan demonstrated bone metastases that were clinically asymptomatic. Treatment was commenced with ICI 118630 s.c. daily injections for 3 months. At this time the MDP bone scan showed deterioration and tamoxifen was substituted. Three months later the bone scan was stable, the local disease improved and serum alkaline phosphate had decreased. She was scored as a tamoxifen responder.

Gonadotrophin data: Pre-treatment serum LH/FSH concentrations were elevated (16/...), rose after the first injection (+2 h 40/43) but this stimulation was only blunted over the first month (D28 pre-injection <0.7/5.4, +2 h 8.7/12.3). Hormone concentrations were not obtained during the second and third months.

*Patient 7* Presented at age 62 years with an apparently localised breast cancer treated by mastectomy. The ER was 30 and PR 28 fmol mg$^{-1}$. Two years later the patient developed a painful right hip and the MDP bone scan

**Figure 3** (a) Chest X-ray picture of patient 2 prior to therapy. Arrows denote metastases. (b) Chest X-ray – same area as in (a) but after 5 months of therapy.

demonstrated multiple bony metastases. Treatment with radiotherapy to the hip and ICI 118630 monthly depot injections was commenced. After 10 weeks of therapy, the bone scan showed new metastases. Tamoxifen therapy was substituted but 10 weeks later the bone scan showed further bone metastatic progression.

Gonadotrophin data: Pre-treatment serum LH/FSH values were elevated (24/30), showed no stimulation in the early days of therapy and suppressed thereafter (D14, 1/4; D21, <0.7/1.2; D28, <0.7/1; D56, <0.7/4).

*Patient 8* Presented at age 61 with a large left breast carcinoma and asymptomatic but assessable metastases on MDP bone scanning. No ER/PR data were available. Treatment was commenced with ICI 118630 depot injections. The MDP bone scan after 4 months showed progressive metastatic disease and the patient was treated with tamoxifen. After two more months, the MDP bone scan demonstrated further progression.

Gonadotrophin data: Pre-treatment serum LH/FSH values were elevated (10/18), did not rise early (D4, 11/14) and were low at day 14 (2/3) and thereafter completely suppressed (D21, <0.7/1; D28, <0.7/1).

*Patient 9* Presented at age 68 with an apparently localised carcinoma of breast treated by mastectomy. No ER/PR data were available. Two and a half years later pain developed in the pubis and the MDP bone scan showed multiple bone metastases. Radiotherapy to the pubis and ICI 118630 depot injections commenced. After three monthly injections the MDP bone scan showed progression. Tamoxifen therapy was substituted and 3 months later the bone scan showed progression.

Gonadotrophin data: Pre-treatment serum LH/FSH values were elevated (31/32), did not rise early (D6, 22/14) and were suppressed by one month (D29 3/7).

*Unassessable*

*Patient 10* Presented at age 35 and was premenopausal with an apparently localised carcinoma of the breast treated by mastectomy. No ER/PR data were available. Two years later the patient complained of backache. The MDP bone scan did not suggest metastatic disease but nevertheless the patient underwent oophorectomy. At age 45 years, the patient developed axillary nodal recurrence that was biopsy proven (ER 661 and PR 0 fmol mg$^{-1}$). ICI 118630 daily s.c. injections were commenced but response could not be scored as radiotherapy to the area was given.

Gonadotrophin data: Pre-treatment LH/FSH levels were raised (28/46) and showed stimulation after injections (D2, +2 h: >50/>80; D8, >50/43; D21, +2 h 27/33; D28, +2 h 33/32). Gonadotrophin suppression was not achieved by 28 days in this patient.

*Chemical endocrine data*

Gonadotrophin data have been presented on each individual patient. It is clear that the daily s.c. injections have proved less satisfactory at effecting gonadotrophin suppression within the first month of treatment (Table I).

Serum levels of oestradiol, oestrone, progesterone, testosterone, $DHASO_4$, androsternedione, cortisol, prolactin, GH, ACTH were serially monitored in all ten patients before and at several (early, intermediate and late) time points during ICI 118630. These data show no change at any time point.

### Discussion

A preliminary report (Harvey *et al.*, 1981) claimed that 12/31 (40%) of postmenopausal women with metastatic cancer benefited from leuprolide treatment but these authors have never definitively published their studies. However, the results are of great interest because hypophysectomy has been known to be effective therapy in postmenopausal women with metastatic breast cancer and its mode of action has never been satisfactorily explained.

In the non-randomised Guy's Hospital series (Hayward *et al.*, 1970), hypophysectomy proved better than adrenalectomy and Henderson and Canellos (1980) cite anecdotal evidence of a response to hypophysectomy after adrenalectomy – both suggesting the the mode of action was not via the pituitary-adrenal axis.

With the availability of potent long acting LHRH analogues and the discovery that they induce down-regulation of pituitary gonadotrophin receptors, came the opportunity to inhibit the anterior pituitary secretion of gonadotrophins in

**Table I** Gonadotrophin levels at sampling time points during daily s.c. injections of ICI 118630 and during the first month after depot injection.

| *Daily s.c. injection n=6* | | *LH* ($IU\,l^{-1}$)$\pm$*s.d.* | *FSH* ($IU\,l^{-1}$)$\pm$*s.d.* |
|---|---|---|---|
| Pretreatment | | 17±15 | 26±22 |
| Day 1 | Pre | 42±16 | 63±14 |
| | Post | 48±4 | 67±14 |
| 1 week | Pre | 7±5 | 12±11 |
| | Post | 18±20 | 17±15 |
| 3 weeks | Pre | 3±2 | 10±5 |
| | Post | 13±8 | 15±10 |
| 4 weeks | Pre | 4±3 | 9±6 |
| | Post | 11±12 | 13±10 |
| *Monthly depot injection n=4* | | | |
| Pretreatment | | 19±8 | 29±7 |
| 1 week | | 18±4 | 13±1 |
| 3 weeks | | 1±0.2 | 5±6 |
| 4 weeks | | 1±1 | 3±3 |

postmenopausal women with breast cancer without affecting the other anterior pituitary hormones.

It is also possible that circulating pharmacological concentrations of a potent LHRH analogue could theoretically have a direct effect on breast cancer cells if they were to possess LHRH receptors. There are some laboratory data which demonstrate LHRH receptors in breast tumours (Lamberts *et al.*, 1982), and recently Eidine *et al.* (1985) and Miller *et al.* (1985) have described LHRH binding sites in human breast carcinoma cells.

In the study reported here, two postmenopausal women showed an objectively measured and durable remission of the metastatic breast cancer (in bones and lungs respectively) following therapy with the potent LHRH analogue ICI 118630. One of these responses was interpreted from the disappearance of 'hot spots' from the technetium methylene diphosphonate bone scan in the lumbar spine – where plain X-rays and CT scanning had demonstrated metastatic disease. This response on bone scanning does not meet UICC criteria because the UICC do not accept bone scan responses. Profound suppression of gonadotrophin secretion from the pituitary was achieved in both patients and no other anterior pituitary hormone, nor cortisol, oestrogens nor androgens were perturbed, as measured by serial blood levels throughout the treatment period. Seven patients failed to respond but several points must be made which may be relevant, if the mechanism of response to ICI 118630 is effected by suppression of circulating gonadotrophin levels. The daily s.c. injection of ICI 118630 proved less successful in down-regulating pituitary gonadotrophin secretion than the depot formulation. This fact together with the finite period allowed for drug administration by the local ethical committee in the early part of the study meant that, in several patients, the pituitary gonadotrophin secretion still had not been suppressed fully by the time the drug was stopped. This last observation may confound the response to subsequent tamoxifen, a manoeuvre that had been deliberately built into the study design for non-responders to ICI 118630. For example, although two responders to tamoxifen were observed, three patients had not fully suppressed their gonadotrophins with daily s.c. ICI 118630. It is therefore difficult to interpret the tamoxifen cross-over data in this trial.

Five patients with tumours known to be ER receptor positive were entered into this study, but little may be concluded concerning the relationship of ER positivity to the response to LHRH analogues. In patient 1, there was a good response to ICI 118630 in a tumour which was ER/PR rich; the ER/PR status of the other responder's tumour is unknown. In the non-responders with tumours very rich in ER/PR, circulating gonadotrophins were not fully suppressed during the period of study, although they may have been so during the second and third months in patient 6. Both of these patients responded to subsequent tamoxifen. In patient 10 with a very rich ER positive tumour, the metastatic disease became unassessable due to elective radiotherapy. In patient 7 the ER/PR values were modestly raised and she responded to neither ICI 118630 nor tamoxifen. It remains important to determine the relationship of tumour response to LHRH analogues and the ER/PR status and data on cross-over to tamoxifen in failing patients.

The LH/FSH levels were initially raised in both responding patients and, indeed, were high in all patients studied except two: one of these, the oldest patient studied (patient 4 – aged 80 years), had very low pre-treatment gonadotrophin values that were hardly stimulated with daily s.c. 118630 injections and quickly became fully suppressed. If LHRH analogues prove effective therapy in postmenopausal breast cancer and if their mode of action should prove to be mediated through effects on pituitary gonadotrophin secretion, the physiological decline in pituitary gland gonadotrophin hypersecretion with time after the menopause provides another variable for consideration. The other patient with low pre-treatment gonadotrophin values (patient 3) had been subjected to artificial menopause (bilateral oophorectomy) at a relatively young age and it was 16 years following this when she received ICI 118630. In complete contrast to the 80 year old patient, this patient had an enormous response to her first daily injection of ICI 118630 and serum gonadotrophins were still not fully suppressed after 22 daily injections.

In this study, the more recently introduced monthly depot preparation of ICI 118630 proved much more effective than the daily s.c. injection in rapidly and completely suppressing pituitary gonadotrophin secretion. By day 4 there was no gonadotrophin hypersecretion and all four patients showed full suppression by three weeks. It may prove essential to analyse the endocrine and clinical data side by side in a study such as this. Without the endocrine data it is highly probable that different and, perhaps wrong, conclusions would have been drawn with regard to tumour response and ER positivity and the tamoxifen cross-over data.

It is concluded that the long acting, potent LHRH analogue ICI 118630 is capable of inducing remissions in metastatic breast cancer in previously untreated postmenopausal women. The mechanism of the response is unknown but serum concentrations of anterior pituitary hormone (other than gonadotrophin) and sex steroid blood levels are not perturbed. Thus these other hormones are probably not involved in the mechanism of drug action. If the response is mediated by the inhibition of pituitary gonadotrophin secretion then the true response rate may be higher than the 2/9 reported here as not all patients achieved full suppression in this study. It may be pertinent to note that full suppression was documented in both responders reported here. The length of time from the menopause, the pre-treatment LH/FSH levels and the ER/PR concentrations in the tumour may be possible predictive factors of a response. An alternative possibility is that the long acting LHRH analogues are directly influencing breast tumour growth. The cross-over data to tamoxifen, amongst those failing ICI 118630, requires further study.

It is with great pleasure that we thank Imperial Chemical Industries for supplying ICI 118630 (Zoladex) and funding the assays, and in particular the assistance of Dr. R. Milstead, Dr. R. Donnolly, Dr. R. Cotton, Mr. D. Richards and Mr. D. Faulkener. Miss J. Seems kindly typed the manuscript.

### References

AHMED, S.R., BROOMAN, P.J.C., SHALET, S.M., HOWELL, A., BLACKLOCK, N.J. & RICKARDS, D. (1983). Treatment of advanced prostatic cancer with LH-RH-analogue ICI 118630: initial response and hormonal mechanism. *Lancet* **ii**, 415.

ALLEN, J.M., O'SHEA, J.P., MASHITER, K., WILLIAMS, G. & BLOOM, S.R. (1983). Advanced carcinoma of the prostate: treatment with a gonadotrophin releasing hormone agonist. *Brit. J. Med.* **286**, 1607.

BORGMAN, V., HARDT, W., SCHMIDT-GOLLWITZER, M., ADENAUER, H. & NAGEL, R. (1982). Sustained suppression of testosterone production by the luteinizing hormone-releasing hormone agonist buserelin, in patients with advanced prostate carcinoma. A new therapeutic approach. *Lancet* **i**, 1097.

CAMERON, E.H.D., HILLIER, S.G., GRIFFITHS, K. (Eds) (1975). *Steroid Immunoassay*. Alpha Omega Publishing Ltd.

DYAS, J., RIAD-FAHMY, D. & RIAD-FAHMY, D. (1979). A simple robust assay for testosterone in male plasma using a $^{125}$I-radioligand and a solid-phase separation technique. *Annals Clin. Biochem.* **16**, 325–331.

EIDNE, K.A., FLANAGAN, C.A. & MILLAR, R.P. (1985). Gonadotrophin-releasing hormone binding sites in human breast carcinoma. *Science* **229**, 989.

GROOM, G.V. (1977). The measurement of human gonadotrophins by radioimmunoassay. *J. Reprod. Fertil.* **51**, 273.

HARVEY, H.A., LIPTON, A., SANTEN, R.J. & 7 others (1981). Phase II study of a gonadotrophin-releasing hormone analogue (Leuprolide) in postmenopausal advanced breast cancer patients. *Proc. Amer. Assoc. Can. Res/Amer. Soc. Clin. Oncol.* **22**, 444.

HAYWARD, J.L., ATKINS, H.J.B., FALCONER, M.A. & 4 others (1970). Clinical trials comparing transfrontal hypophysectomy with adrenalectomy and with transethmoidal hypophysectomy. In *The Clinical Management of Advanced Breast Cancer*, Joslin, C.A.F. & Gleave, E.N. (eds). p. 50. Alpha Omega Alpha Publ.: Cardiff, Wales.

HENDERSON, I.C. & CANELLOS, G.P. (1980). Cancer of the breast. The past decade. *N. Eng. J. Med.* **302**, 17 & 78.

KLIJN, J.G.M, & DE JONG, F.J. (1982). Treatment with a luteinising-hormone releasing hormone analogue (Buserelin) in premenopausal patients with metastatic breast cancer. *Lancet* **i**, 1213.

LAMBERTS, S.W.J., TIMMERS, J.M., OOSTEROM, R., VERLEUN, T., ROMMERTS, F.G. & DE JONG, F.H. (1982). Testosterone secretion by culture arrheno-blastoma cells: suppression by a luteinizing hormone-releasing hormone agonist. *J. Clin. Endocrinol. Metab.* **54**, 450.

MILLER, W.R., SCOTT, W.N., MORRIS, R., FRASER, H.M. & SHARPE, R.M. (1985). Growth of human breast cancer cells inhibited by a luteinising hormone releasing hormone agonist. *Nature*, **313**, 231.

NICHOLSON, R.I., WALKER, K.J., DAVIES, P. & 7 others (1984). Use and mechanism of action of the LHRH agonist ICI 118630 in the therapy of hormone sensitive breast and prostate cancer. Raven Press.

READ, G.F., RIAD-FAHMY, D. & DYAS, J. (1963). Immunoassays employing magnetisable, solid-phase anti-steroid sera. In *Immunoassays for clinical Chemistry*, Hunter, W.M. & Corrie, J.E.T. (eds), p. 63. Churchill Livingstone.

RIAD-FAHMY, D., READ, F.G., GASKELL, S.J. & 2 others (1979). A simple direct radioimmunoassay for plasma cortisol, featuring a $^{125}$I-radioligand and a solid-phase separation technique. *Clinical Chemistry* **25**, 665.

SMITH, M.R., RUDD, B.T., SHIRLEY, A. & 4 others (1975). A radioimmunoassay for the estimation of serum dehydroepiandosterone sulphate in nominal and pathological sera. *Clin. Chim. Acta* **65**, 5.

TOLIS, F., CHAPDELAINE, A., ROBERTS, K. & 4 others (1981). In *Endocrinological Cancer*, Adelcreutz, H. *et al* (eds). p. 79. Excerpta Medica Series.

TOLIS, F., ACKMAN, D., STELLOS, A. & 5 others (1982). Tumour growth inhibition in patients with prostatic carcinoma treated with luteinizing hormone-releasing hormone agonists. *Proc. Natl Acad. Sci. USA* **79**, 1658.

WALKER, K.J., NICHOLSON, R.I., TURKES, A.O. & 5 others (1983). Therapeutic potential of the LH-RH agonist ICI 118630, in the treatment of advanced prostatic carcinoma. *Lancet* **ii**, 413.

WAXMAN, J.H., WASS, J.A.H., HENDRY, W.F. & 3 others (1983). Treatment with gonadotrophin releasing hormone analogue in advanced prostatic cancer. *Brit. Med. J.* **286**, 1309.